# Association between daily variations in the levels of atmospheric O_3_, PM_2.5_, and NO_2_ and the frequency of hospital visits due to respiratory diseases and hypertension in Mexico City using Generalized Additive Mixed Models

**DOI:** 10.3389/fpubh.2025.1593285

**Published:** 2025-09-02

**Authors:** Elizabeth Vega, Alejandro Ruiz-Olivares, Yosune Miquelajauregui, Regina Rentería-Campos, Lindsay Bramwell, Romain Bernard M. Feytmans, Anil Namdeo, Juan Carlos Núñez-Enríquez, Jane A. Entwistle, Mónica Jaimes-Palomera, Nancy Minerva Torres-Rojas, R. Alberto Rascón-Pacheco, David A. Duarte-Rodríguez, Richard J. Q. McNally, Jimena García-Burgos, Cinthia Gabriela Resendiz-Martinez, Ángel Fragoso-Chino, Louise Hayes, Antonio Portas, Juan Manuel Mejia-Arangure

**Affiliations:** ^1^Instituto de Ciencias de la Atmósfera y Cambio Climático, Universidad Nacional Autónoma de México (UNAM), Mexico City, Mexico; ^2^Department of Soil Sciences, Colegio de Postgraduados, Montecillo, Mexico; ^3^Laboratorio Nacional de Ciencias de la Sostenibilidad, Instituto de Ecología, Universidad Nacional Autónoma de México (UNAM), Mexico City, Mexico; ^4^Facultad de Medicina, Universidad Anáhuac México Sur, Mexico City, Mexico; ^5^Department of Geography and Environmental Sciences, Northumbria University, Newcastle upon Tyne, United Kingdom; ^6^Unidad de Investigación Médica en Epidemiología Clínica, UMAE Hospital de Pediatría Siglo XXI, Instituto Mexicano del Seguro Social (IMSS), Mexico City, Mexico; ^7^Dirección de Monitoreo de Calidad del Aire, Secretaria del Medio Ambiente, Gobierno de la Ciudad de Mexico, Mexico City, Mexico; ^8^Coordinación de Vigilancia Epidemiológica, Instituto Mexicano del Seguro Social, Mexico City, Mexico; ^9^Unidad de Educación, Investigación y Políticas de Salud, Instituto Mexicano del Seguro Social, Ciudad de Mexico, Mexico; ^10^Population Health Sciences Institute, Newcastle University, Newcastle upon Tyne, United Kingdom; ^11^Laboratorio de Ecología, Unidad de Biología de la Conservación, Parque Científico y Tecnológico de Yucatán, Universidad Nacional Autónoma de México (UNAM), Mérida, Mexico; ^12^Análisis y Modelación de Calidad del Aire, Secretaría de Medio ambiente de la Ciudad de México, Mexico City, Mexico; ^13^Department of Mathematics, Physics and Electrical Engineering, Northumbria University, Newcastle upon Tyne, United Kingdom; ^14^Genómica del Cáncer, Instituto Nacional de Medicina Genómica, Mexico City, Mexico

**Keywords:** hypertension, respiratory diseases, particulate matter 2.5 μm (PM) 2.5, Mexico City (CDMX), air pollution, asthma, children, aging

## Abstract

**Background:**

Environmental pollution is a significant public health issue in Mexico City. Patients with respiratory or cardiovascular diseases such as asthma or hypertension often experience exacerbations triggered by environmental factors. This retrospective time series epidemiological study analyzed the association between daily air pollution levels and the numbers of medical visits for exacerbations of cardiorespiratory diseases.

**Methods:**

Records from primary, secondary, and tertiary hospitals of the Mexican Institute of Social Security (IMSS) were reviewed between 2017 and 2019. Air quality data, including PM_2.5_, PM_10_, O_3_, and NO_2_ concentrations were collected hourly from air quality monitoring stations at fixed sites. To fit the models and

take into account temporal autocorrelation and the complex non-linear relationships between pollutants and cardiorespiratory diseases, Generalized Additive Mixed Models (GAMM) were applied.

**Results:**

PM_2.5_, O3, and NO2 exposure showed a strong association with an increase in visits for upper respiratory diseases. Age was a relevant factor: individuals aged between 19–35 years were the most affected, whereas those aged 66–100 years were least affected. An effect on the weekdays was identified, with peaks in visits on Mondays and decreases on Saturdays. Also, seasonally, results showed an increase in October and declines in August. Regarding hypertensive diseases, only PM_2.5_ exhibited an association. The models confirmed the link between air pollution levels and respiratory disease exacerbations, highlighting key public health implications.

**Conclusion:**

This study provides strong evidence that short term exposure to elevated concentrations of atmospheric pollutants, specifically PM_2.5_, O3, and NO2, are significantly associated with an increase in medical visits for upper respiratory diseases, asthma, and hypertension in Mexico City. By employing Generalized Additive Mixed Models and analyzing health records from IMSS-affiliated hospitals between 2017 and 2019, we observed that the effects of air pollution varied by disease, age group, season, and day of the week.

## Introduction

PM_2.5_ consists of a complex mixture of chemical compounds, including heavy metals, polycyclic aromatic hydrocarbons, organic carbon, trace elements, and secondary ions such as sulfates and nitrates. A study conducted across multiple regions in Asia revealed that a significant proportion of PM_2.5_ mass originated from organic matter, primarily associated with biomass burning and from secondary inorganic aerosols, predominantly sulfates. The main contributors identified included vehicular emissions, biomass combustion, inorganic ions, and mineral dust. Additionally, elevated levels of nitrates and heavy metals were detected. These pollutants were particularly prevalent in samples collected from urban, agricultural, and industrial areas. Key contributors include traffic emissions, construction activities, open burning, and industrial processes. This evidence reinforces the predominant role of anthropogenic sources in PM_2.5_ formation. Furthermore, the study demonstrated that meteorological variables, including temperature, humidity, and wind speed, significantly influence both the composition and ambient concentration of PM_2.5_ (1).

Although PM_2.5_ is a key contributor to the pathophysiological mechanisms underlying various respiratory and cardiovascular diseases, it rarely exists alone. Instead, it frequently interacts with other atmospheric pollutants, leading to complex chemical dynamics. For instance, carbon monoxide (CO) and sulfur dioxide (SO_2_) can undergo multiple oxidation processes in the atmosphere, resulting in the formation of hydroxyl radicals, ozone (O3), and sulfate ions (SO_4_^2−^). These reactive species can modify the chemical composition of the air and influence PM_2.5_ concentrations. Evidence shows that when CO levels exceed 3.5 μg/m3 and SO_2_ surpasses 6.0 μg/m3, there is a significant increase in PM_2.5_ concentrations (2). Furthermore, a separate study analyzing the interaction of these pollutants found that nitrogen dioxide (NO_2_) alone was significantly associated with respiratory diseases. However, synergistic effects between NO_2_ and PM_2.5_ were linked to a higher incidence of circulatory diseases, while combined exposure to NO_2_ and SO_2_ was associated with increased respiratory morbidity (3).

International studies have extensively explored the link between respiratory and cardiopulmonary diseases and air pollution. This research consistently shows that exposure to pollutants like particulate matter (PM_2.5_ and PM_10_), nitrogen dioxide (NO_2_), and ozone (O_3_) contributes to the development and exacerbation of respiratory conditions, including asthma, chronic obstructive pulmonary disease (COPD), and lung cancer. Beyond respiratory diseases, air pollution is strongly linked to cardiopulmonary conditions, such as ischemic heart disease, heart failure, arrhythmias, and stroke. Air pollution affects cardiopulmonary health through oxidative stress, inflammation, endothelial dysfunction, and autonomic imbalance. All these factors contribute to the progression of atherosclerosis, thrombosis, and, ultimately, cardiovascular events (4–6).

### Impact on cardiovascular health

Air pollution is actually ranked as the 4th highest risk factor for cardiovascular mortality, attributing more deaths to it than high LDL cholesterol, high body-mass index, physical inactivity, or alcohol use (7, 8). From a pathophysiological standpoint, to PM_2.5_ exposure is associated with abnormal activation of the hemostatic system, which can lead to thrombus formation (7). PM_2.5_ also promotes vascular inflammation and endothelial cell injury contributing to coronary and carotid atherosclerosis. Furthermore, it can trigger atherosclerotic plaque rupture by activating metalloproteinases (9). A study demonstrated that a 10 μg/m3 increase in PM_2.5_ was associated with an 11% increase in cardiovascular mortality (3). Another cohort study evaluated the long-term effects of PM_2.5_ exposure and found a positive association between long-term exposure to PM_2.5_ and stroke incidence (10).

Although the physiopathology of asthma is not well known, it is suggested that air pollution promotes the release of epithelial cytokines, driving TH2 responses, which induce oxidative stress and produce proinflammatory cytokines, disrupting of the airway epithelial barrier (11). Among environmental factors, fine particulate matter (PM_2.5_) stands out, given its potential to trigger inflammatory signaling and induce oxidative stress (12). Long-term air pollution exposure has been associated with increased hospital admissions, reduced lung function, and a heightened risk of premature death. Large-scale cohort studies across North America and Europe to Asia consistently show that individuals living in areas with higher pollution levels face an elevated risk of developing these diseases. For instance, research in Europe and the United States have found a clear correlation between elevated levels of PM_2.5_ and increased mortality from cardiovascular and respiratory diseases. Moreover, studies in developing countries, where air pollution levels are often higher due to rapid industrialization and urbanization, have further underscored air pollution's global health burden.

These findings highlight the urgent need for policies to reduce emissions and improve air quality to protect public health (7, 8, 10, 12, 13).

In Mexico City, the location and the orography also contribute to worsened air pollution. For example, the highest O_3_ concentrations are more frequent in the south of the urban area. Conversely, the PM_2.5_ maximum concentrations are generally observed in the city's north. NO_2_ levels peak in the north and center, correlating with heavier vehicular traffic (14).

This study used Generalized Additive Mixed Models (GAMMs) to fit the frequency of respiratory and cardiovascular hospital visits to air pollutant concentrations in Mexico City. GAMMs consider temporal autocorrelation and the complex non-linear relationships between pollutants and upper respiratory and cardiovascular diseases.

Generalized Additive Mixed Models (GAMM) are an advanced extension of Generalized Additive Models (GAM) and Generalized Linear Mixed Models (GLMM) (15). Their primary purpose is to analyze complex relationships in data, especially in time-series studies, such as the present study, where assumptions of linearity or independence may not be met (15, 16).

We employed GAMM to analyze the association between daily atmospheric pollutant levels and the frequency of hospital visits. The choice of GAMM is due to their design to capture temporal autocorrelation and the non-linear relationships that exist among pollutants, meteorological factors, and health outcomes. Furthermore, GAMM possess the ability to handle heterogeneity in terrain and meteorological conditions and can include random effects (15).

Smoothing functions or splines are an advantage of GAMM, as they allow for the modeling of these non-linear relationships without assuming a rigid parametric form (such as a straight line or a simple polynomial) (15, 16).

## Materials and methods

### Study area

**Figure 1:**
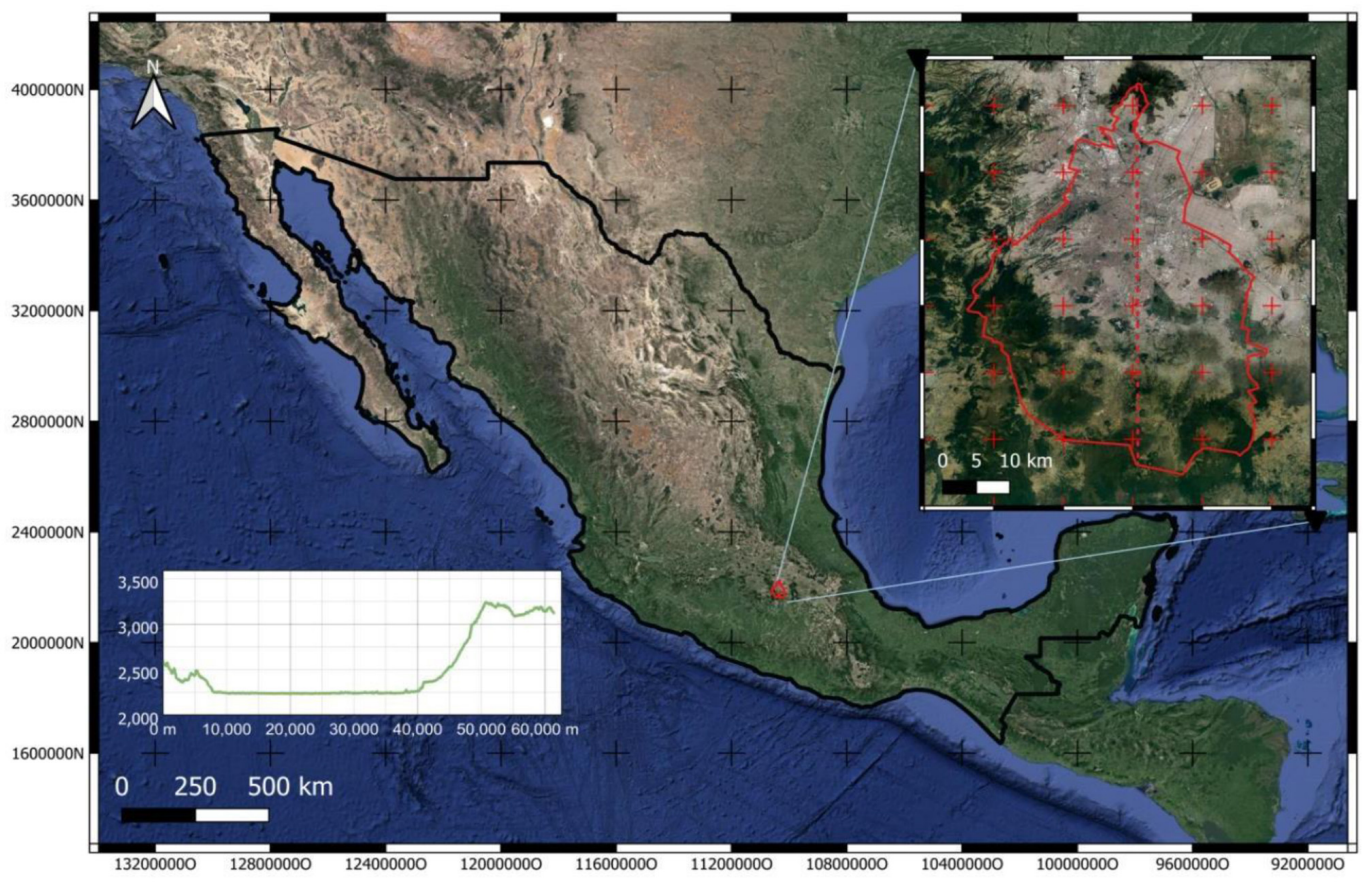
Mexico City location and landscape. The black polygon is the Country, and the red polygon is Mexico City. The plot is the elevation profile of the red dashed line drawn in the city (north to south).

The Metropolitan Zone of the Valley of Mexico is located on one of the central plateaus of the country (Figure 1), with an altitude ranging between 2,223 and 3,510 meters above sea level. It is relevant that the sectors and neighborhoods inhabited at the highest altitude are more than 3,000 meters above the sea level in the south of the city because this creates complex and irregular meteorological behavior. Figure 1 shows an elevation profile from the north to the south of the city. It is possible to observe the high-altitude variation (approximately 1,500 meters in just 60 km), and a plateau can be observed at the south of the urban territory (center of Mexico City). The southern part of Mexico City (53% of the geographical demarcation) is designated as a conservation zone, primarily consisting of mountainous terrain. The climate is temperate, with rainfall in summer (from 600 mm per year in the northern plains to more than 1,500 mm in the southern mountains), with a wet season in summer, from May to October, with a maximum from June to September; the rest of the year is dry, warm from March to May and cold from November to February. During the winter, there can be frost and even occasional snowfall in the upper parts of the surrounding mountains. The months of the dry seasons of the year (warm-dry and cold-dry) are the ones with the highest levels of air pollution. Additionally, the prevailing winds tend to be weak, especially during the dry seasons of the year, with little capacity to carry pollutants, a condition that, together with the direction of the prevailing winds (from the north in winter and northeast in summer), causes that in their displacement many times they are not able to overcome the mountains located in the opposite position, which surround the city on three of its flanks (south, east and west). These situations configure frequent thermal inversions, especially in winter, which aggravate the atmospheric pollution conditions of this urban agglomeration.

In the Air Quality and Health Annual, the Environmental Authorities in Mexico City reported that during December 2023, the concentration of PM_2.5_ ranged between 60 μg/m^3^ and 108 μg/m^3^, showing that the values were above the Mexican National Ambient Air Quality Standards (NAAQS) (7). In addition, the concentrations of O^3^ in the downtown area of the city recorded in that period were maximum average concentrations of 111 ppb and minimums of 14 ppb, whereas NO^2^ had a maximum of 51 ppb and a minimum of 17 ppb (7). Moreover, the Mexican National Standards (NOMs) establish an average concentration of 24-hr of 45 μg/m^3^ for PM_2.5_, 70 ppb for O_3_, and 210 ppm for NO_2_ (6).

### Retrospective time series analysis

#### Hospital visits data

Data on disease-related hospital visits were obtained from the Mexican Institute of Social Security (IMSS) Registration Department. This data encompassed all public hospitals and clinics (primary, secondary, and tertiary care levels) affiliated with IMSS, spanning from 2017 to 2019. Starting in 2017, the IMSS implemented a system capable of recording daily medical care, which is why data from earlier years, reported monthly, could not be included in this analysis. The dataset included the number of hospital admissions according to asthma, upper respiratory diseases, hypertension, and myocardial cardiovascular diseases. Diseases were classified using the International Classification of Diseases (ICD-10) codes in two main categories: acute respiratory diseases (comprising upper respiratory tract infection, bronchitis, bronchiolitis, and/or pneumonia) and asthma. Demographic information, including age and gender, is included. We focused our analysis on children under 5 years old and adults aged 19 years and older. These age groups were selected due to their heightened vulnerability and higher incidence of specific conditions: acute respiratory diseases in children, and acute myocardial infarctions, hypertensive crises, and chronic obstructive pulmonary diseases in adults.

#### Air pollution data

Hourly air quality data for the period 2017–2019 for PM_2.5_, O_3_, and NO_2_ were obtained from ten fixed air quality monitoring stations in Mexico City and the metropolitan area. Only those stations that provided data for at least 80% of the days and the time period covered were included. Humidity and temperature data were obtained from the Mexico City meteorological network stations. The air quality data was generated by the Automatic Network of Atmospheric Monitoring System of Mexico City, established by Mexico City's Environmental Agency (SEDEMA, according to its Spanish acronym) (14). The pollutant averaging times used for the study correspond to current air quality standards in Mexico established by the Mexican government and outlined in the official Mexican regulations (“Norma Oficial Mexicana”) (NOM-025-SSA1-2014, NOM-020-SSA1-2014, NOM-023-SSA1-1994) (17–19) which are, at the same time, congruent with health studies and are listed below:

**O**_**3**_: Daily maximum of 8 h moving average (ppb).
**PM**_**2.5**_: 24 hours average (μg/m^3^).
**NO**_**2**_: Daily maximum of 1-h concentration (ppb).

Data were included in the analysis if they met an 80% completeness threshold. This ensured that data for each pollutant were calculated for at least 17 h per day. To maximize the number of observations for the analysis, missing data were imputed using a moving average method that incorporated both previous and succeeding available data points. The maximum number of consecutively missing values permitted for imputation was set to six to avoid unreliable estimations over extended periods (see Supplementary Figure 1).

Pollutant concentrations were assigned to hospitals based on the spatial representativeness of each air quality monitoring station. Representativeness areas were defined in a study by SEDEMA (14) which evaluated land use, emission sources, and environmental conditions surrounding each station It follows the criteria recommended by the U.S. Environmental Protection Agency (EPA) in Title 40 of the Code of Federal Regulations (CFR 40). For each hospital, pollutant levels were assigned from the nearest monitoring station whose representativeness buffer included the hospital.

**Figure 2:**
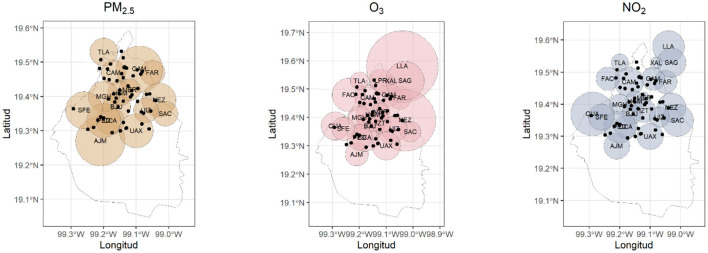
Spatial representativeness of the PM_2.5_, O_3_, and NO_2_ monitoring stations (circles) and the hospitals (black dots) considered in the study.

To avoid misclassification from distant monitoring stations, a maximum distance threshold was applied. Only hospitals located within 5 km of an eligible station were assigned pollutant data. This strategy is justified by the findings of the SEDEMA study. Where the mean representativeness radius across all pollutants was 5,006 meters, and 5,346 meters specifically for PM_2_.5, O3, and NO_2_. Thus, a 5 km threshold was selected as a limit to ensure that assigned concentrations reflected local exposure conditions while preserving adequate spatial coverage. The hospital's location and the spatial representativeness of monitoring stations are shown in Figure 2.

The spatial scale of the representativeness of an atmospheric monitoring station defines the geographic area whose air quality conditions can be approximated by the measurements from that station. This scale varies depending on the type of pollutant, the characteristics of the emission source, the topography, and the weather patterns.

The representativeness of the stations is urban and extends up to a 5 km radius, representing population exposure, general and background conditions, pollutant transport, and their impact on wellbeing.

A 5 km radius around an air quality monitoring station is commonly used as a guideline to assess the representativeness of the station's data. This limit is based on the usual spatial variation of air pollutants and the practical constraints of monitoring networks. It indicates that within this radius, the air quality is likely similar enough for the station's measurements to reflect the area's conditions.

### Statistical analysis

Generalized Linear Mixed Models (GLMM) with Poisson regression were used to fit the time series of selected medical events. The method was adapted from that described by Tadano et al. (20). Poisson regression was chosen because the outcome variable, daily counts of medical events, is a non-negative integer count variable that follows a Poisson distribution. Poisson distribution is commonly used in time series studies of environmental epidemiology to model the occurrence of events, such as hospital admissions or emergency visits over time. These time series were related to daily concentrations of air pollutants (derived from the hourly observations), controlling for temporal trends, weather indicators, day of the week, and epidemics. Likewise, the average daily (2017–2019 analysis) relative humidity and temperature and the season of the year were controlled. In addition, for the daily analysis (2017–2019), the day of the week and holidays were controlled.

The relative risks of a hospital admission associated with an increase of 10 units (μg m-3) of PM_10_ and an increase of 5 units of PM_2.5_ and NO_2_ were calculated. In addition, the relative risks for an increase in the interquartile range of contaminants were calculated. For the daily analysis, delay times from 0 to 7 days were considered. The Akaike Information Criterion (AIC) was used to identify the best-fitting model. Analyses were performed separately for adults (over 18 years) and for children (under 5 years).

GAMMs helped to fit the hospital ad into account temporal autocorrelation and the complex non-linear relationships between pollutants and upper respiratory diseases. GAMMs models can handle the complex variations in Mexico City that are caused mainly by the orography shown in Figure 1. GAMMs extend the capabilities of generalized additive models, including random effects, in addition to the parametric additive terms. Hence, the splines can be replaced by random effects as Equation 1 shows (21).


(1) $$\begin{array}{l} g\left(\mu_{it}\right)=x_{it}^{T}\beta \;+\;\overset{m}{\underset{j=1}{\sum }}\alpha_{j}\left(u_{itj}\right)+\;z_{it}^{T}b_{i}=\;\eta_{it}^{par}+\;\eta_{it}^{add}+\;\eta_{it}^{rand} \end{array}$$

Where:

*g*(μ_*it*_) *Is the link function (log) connecting the mean response* μ_*it*_ *(expected hospital admissions for location*_*i*_ *at time*_*t*_*) to the predictors*

$x_{it}^{T}\beta$ = $\eta_{it}^{par}$ *is a linear parametric term with parameter* ${\beta^{T}=\;\beta }_{0}+\beta_{1},\ldots ,\;\beta_{p}$

$\overset{m}{\underset{j=1}{\sum }}\alpha_{j}\left(u_{itj}\right)$ = $\eta_{it}^{add}$ *is an additive term with functions* α_(1 )_, …, α_*m*_

$z_{it}^{T}b_{i}=\eta_{it}^{rand}$ contains the random effects.

In addition, the use of different distributions can be complemented by modeling residual autocorrelation through Auto Regressive Moving Average (ARMA) functions (21).

For model validation and selection, the Akaike Information Criteria (AIC) was used, where its lowest values indicate better model fitting. The AIC is defined in Equation 2:


(2) $$\begin{array}{l} AIC=2k-2\;ln\;ln\left(\hat{L}\right) \end{array}$$

Where K is the number of estimated parameters in the model and $\hat{L}$ is the maximum value of likelihood function of that model.

## Results

**Figure 3:**
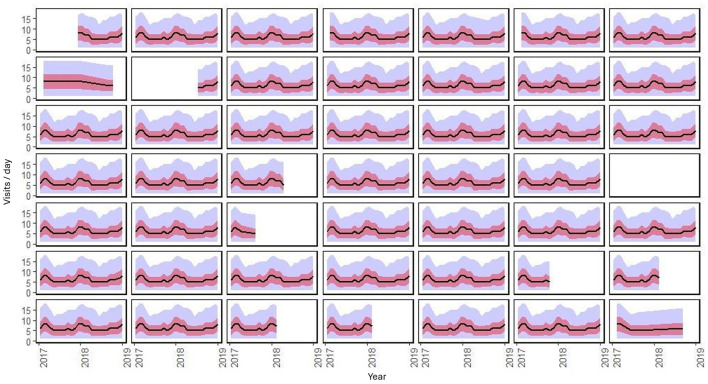
” Temporal behavior by month of the 49 hospitals during the period of analysis. The black line is the average daily visits, the red ribbon is the standard deviation, and the blue ribbon is the minimum and maximum.

The monthly behavior of visits had a similar trend among hospitals, with a peak in 2018 where seasonal behavior can be observed. However, part of the information about ten hospitals was missing. In this case, there was no imputation because of a lack of randomness in those missing points, which consist of continuous periods of time (Figure 3).

**Figure 4:**
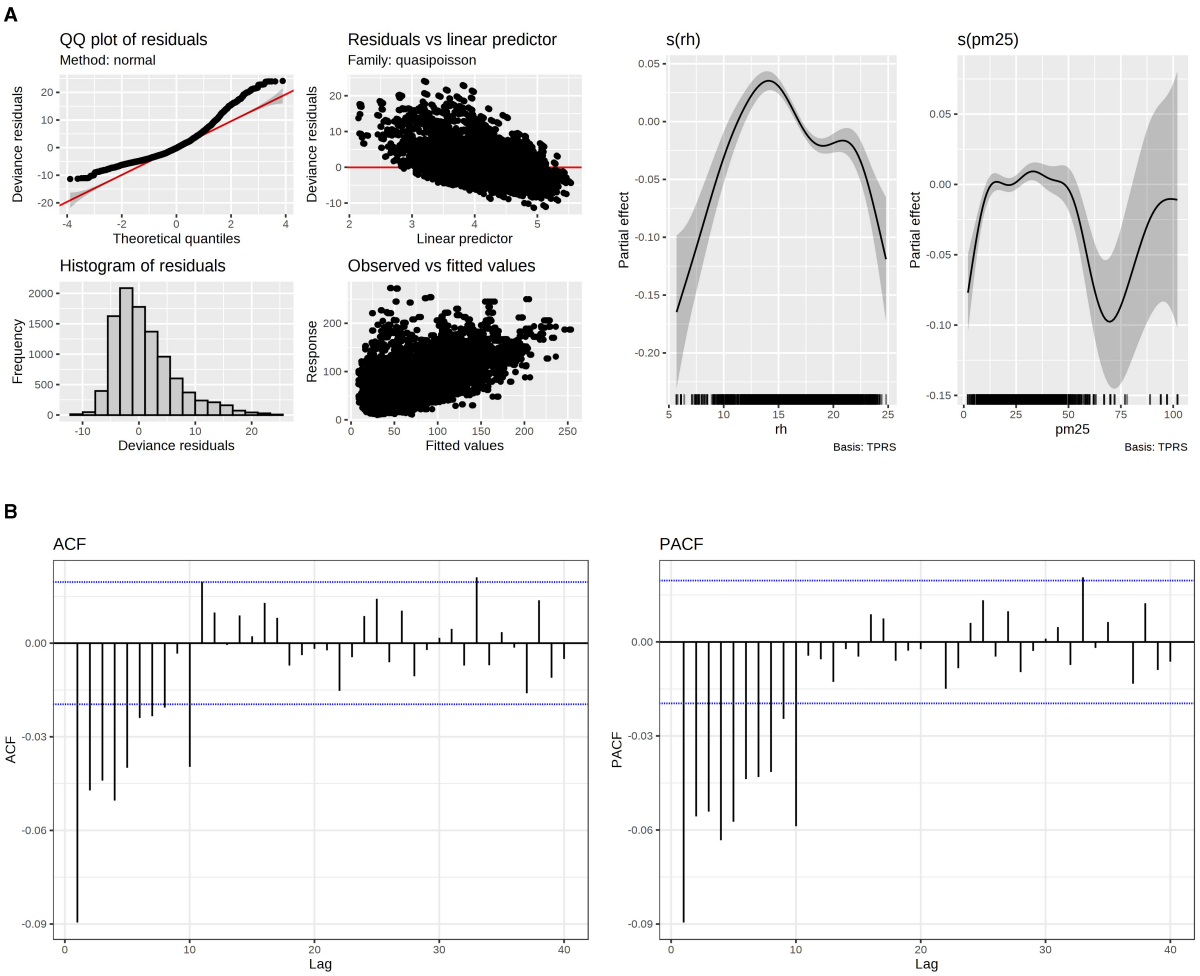
**(A)** Model fitting evaluation of upper respiratory diseases (left) and PM_2.5_ and RH smoothers. **(B)** Autocorrelation Function (ACF) and Partial Autocorrelation Function (PACF) for upper respiratory diseases.

The models that exhibited the best fit were the ones that accounted for all three pollutants as factors that influence the frequency of disease-related visits. The results showed a significant association between the number of visits for upper respiratory diseases and the three pollutants under consideration. Specifically, O_3_ and NO_2_ had a positive effect on the outcome measure, while PM_2.5_ exhibited a non-linear impact requiring a smoother. Similar behavior was found in relative humidity, with different percentages having different effects on the number of visits. From a range between 0 to 12%, it had a negative partial effect. Above 12%, the effect is positive, and then it inverts the effect from 17%. These findings are supported by the results presented in Table 1 and Figure 4.

**Table 1:** Result of the best fitting model for upper respiratory diseases.

**Variable**	**Estimate**	**(Pr > t) < 0.05**	**Visits**
O_3_	4.80E-04	^*^	+
NO_2_	2.16E-04	^*^	+
Age 66–100	3.30E+00	^*^	+
Age 51–65	3.62E+00	^*^	+
Age 36–50	3.89E+00	^*^	+
Age 0–5	4.53E+00	^*^	+
Age 19–35	4.66E+00	^*^	+
Monday	2.13E-01	^*^	+
Tuesday	9.14E-02	^*^	+
Wednesday	6.49E-02	^*^	+
Thursday	1.07E-02	NS^*^	+
Friday	5.30E-03	NS	+
Saturday	−2.52E-02	^*^	-
August	−2.54E-01	^*^	-
			NS
July	−2.16E-01	^*^	-
June	−1.48E-01	^*^	-
May	−9.36E-02		-
November	1.23E-01	^*^	+
September	1.23E-01	^*^	+
March	3.57E-01	^*^	+
January	4.26E-01	^*^	+
December	4.33E-01	^*^	+
February	4.70E-01	^*^	+
October	4.82E-01	^*^	+
Lag day1	4.00E-02	^*^	+
Lag day 2	9.97E-03	^*^	+

The sign in the visits column means increment (+) or reduction (–) in the number of visits. NS, Statistically non-significant (*p* > 0.05), R2: 0.69. Smoother: s(rh), s(pm25). ^*^p-value < 0.05.

Regarding age, individuals aged between 19–35 and 66–100 years were most and least affected, respectively. The analysis also showed that Monday was associated with an increase in the number of visits, whereas Saturday was associated with a reduction.

Regarding seasonal effects, October had the most substantial increase in the number of visits, whereas August had a negative impact. Regarding day lags, a significant positive effect was found on the first 2 days of exposition.

Regarding the ACF and PACF plots, the residuals demonstrated minimal correlation, with values falling inside the autocorrelation thresholds. This indicates that the correlations are likely due to random chances and are not meaningful. This was accomplished by incorporating p = 1 and q = 1 terms into the correlation matrix within the GAMM model.

Regarding fitting, the Poisson distribution was more suitable for the data. However, there is still information that the model couldn't fit.

Relative risk for O_3_ and NO_2_ is defined as:


$$\begin{array}{r} RR_{O_{3}}=\exp\;\left(4.8E-04\right)\;=1.0\;visits\;per\;O_{3}\;unit\\ RR_{{NO}_{2}}=\exp\;\left(2.16E-04\right)\;=\;1.0\;visits\;per\;NO_{2}\;unit \end{array}$$

Which means that if there is an interval of 50 ppb and then there is an increment to 60 ppb for O_3_, the visits will increase in 10. In the same way, an increase in NO_2_ concentration from 60–70 ppb for NO_2_, the visits due to upper respiratory diseases will be increased in 10.

**Figure 5:**
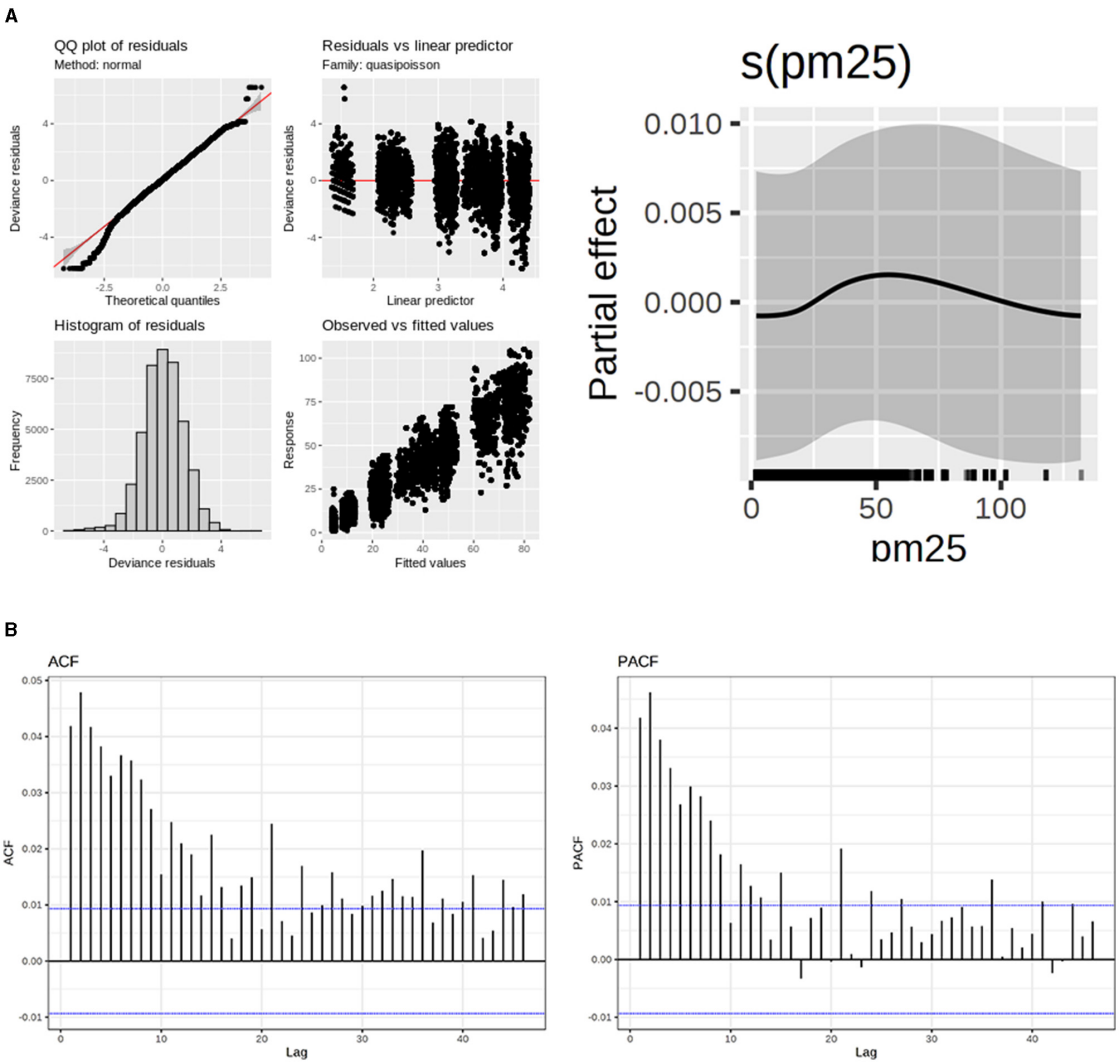
**(A)** Model fitting evaluation of hypertensive diseases. **(B)** Autocorrelation Function (ACF) and Partial Autocorrelation Function (PACF) for hypertensive diseases.


On the other hand, PM_2.5_ could not be interpreted only with one relative risk. This can be observed in the smoothers for this pollutant (Figure 5). For instance, in 37 μg/m^3^ and 63 μg/m^3^, there are the maximum and minimum relative risks respectively:

$$\begin{array}{l} {RR_{PM}}_{2.5}\left(37\;\mu g/m3\;\right)=\exp\;\left(0.02\right)\;=1.02\;visits\;per\;PM_{2.5}\;unit\\ {RR_{PM}}_{2.5}\left(63\;\mu g/m3\right)=\exp\left(-0.08\right)=0.92\;visits\;per\;PM_{2.5}unit \end{array}$$

The smoother plots provide a visual representation of the non-linear relationship between PM_2.5_ concentrations and the relative risk of upper respiratory diseases. These plots are generated using GAMMs, which allow for the inclusion of random effects and parametric additive terms. This modeling approach is particularly useful in capturing the complex interactions between pollutants and health effects.

The non-linear pattern observed in the smoother plots highlights the importance of considering varying pollutant levels rather than relying on a single RR value. For example, at lower concentrations of PM_2.5_, the RR may increase, indicating a higher risk of health issues. However, as the concentration increases, the RR may decrease, suggesting a different impact on health effects.

Regarding hypertensive diseases, the effect of pollutants was only found for PM_2.5_ as smoother. However, the partial effect was nearly zero (Figure 5). A positive effect was observed in older people about age. Table 2 shows that, in older people, visits to hospitals due to hypertensive diseases were more common. People aged 19 to 35 years were the least affected by hypertensive disease. Regarding the day of the week, all days have a positive and similar effect on the visits, except Saturday, which had a low and adverse effect on that variable. Regarding the year's seasonality, only January, May, and June had statistically significant effects, negatively influencing the visits. The impact of lag was found for day 1. The model could fit the data with an ARMA correlation matrix of p = 1 and q = 1 for AR and MA components.

**Table 2:** As a result of the best model for hypertensive diseases, the sign in the visits column means an increment (+) or reduction (-) in the number of visits.

**Variable**	**Estimate**	**(Pr > t) < 0.05**	**Visits**
Age 19–35	1.81	^*^	+
Age 36–50	2.87	^*^	+
Age 51–65	3.40	^*^	+
Age 66–100	3.83	^*^	+
Monday	0.66	^*^	+
Tuesday	0.66	^*^	+
Wednesday	0.68	^*^	+
Thursday	0.64	^*^	+
Friday	0.65	^*^	+
Saturday	0.03	^*^	-
August	−0.02	NS	-
July	−0.05	NS	-
June	−0.06	^*^	-
May	−0.07	^*^	-
November	−0.02	NS	-
September	−0.01	NS	-
March	−0.02	NS	-
January	−0.08	NS	-
December	−0.18	^*^	-
February	−0.02	NS	-
January	−0.08	^*^	-
October	0.09	NS	+
Lag day 1	0.38	^*^	-

NS, Statistically non-significant (*p* > 0.05), R^2^: 0.87. Smoother: s(pm25). ^*^p-value < 0.05.

The relative risk for PM_2.5_ remains constant throughout the entire range of data examined in the study. This is expressed as:


$$\begin{array}{l} {RR_{PM}}_{2.5}=\exp\;\left(0\right)\;=1.0\;visits\;per\;PM_{2.5}\;unit \end{array}$$

**Figure 6:**
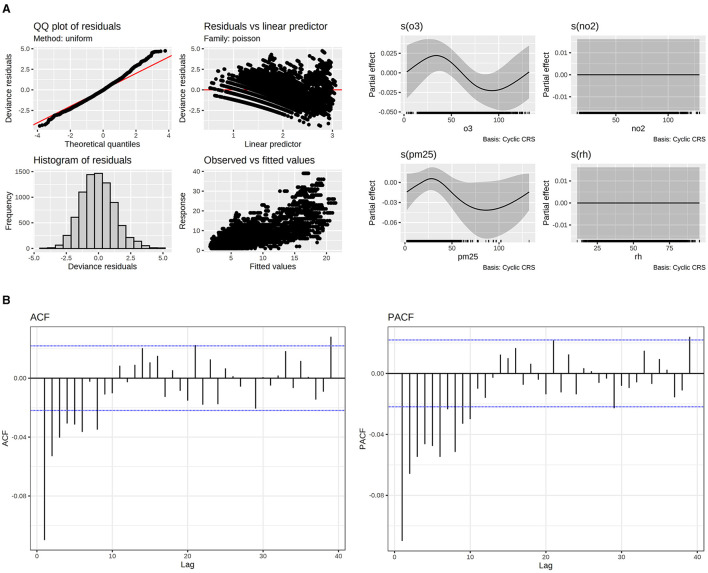
**(A)** Model fitting evaluation of asthma. **(B)** Autocorrelation Function (ACF) and Partial Autocorrelation Function (PACF) for asthma.

The group most affected by asthma was those aged 36–50 years, in contrast to the least affected group of hypertensive diseases being those aged 66–100 years. All days of the week positively affected the number of visits, while PM_2.5_ and O_3_ had significant non-linear effects, exhibiting a cyclic pattern. This behavior refers to the recurring patterns observed in the effects of PM_2.5_. These patterns can be visualized using smoother plots shown in Figures 5, 6, which show how the RR varies with different concentrations of the pollutant. Although Saturday showed a negative effect, it was not substantial. Seasonality effects were only observed in January and February, negatively impacting the number of visits. Regarding lags, all days had adverse effects, except for days 4 and 10 which showed positive effects. Autocorrelation in the residuals was negligible based on ACF and PACF plots, indicating that no significant temporal information remained. This was verified using ARMA terms (q = 1, p = 1) incorporated into the correlation matrix of the GAMM.

For O_3_ and PM_2.5_ relative risks. They can be only interpreted in terms of the partial effects in their smoother (Table 3). Figure 6 shows the following minimum and maximum relative risks for PM_2.5_ in 87 and 37 respectively:


$$\begin{array}{r} {RR_{PM}}_{2.5}\left(37\;ug/m3\;\right)=\exp\left(0.025\right)=1.02\;visits\;per\;PM_{2.5}\;unit\\ {RR_{PM}}_{2.5}\left(87ug/m3\right)=\exp\left(-0.025\right)=0.97\;visits\;per\;PM_{2.5}unit \end{array}$$

In the case of O_3_ the maximum and minimum relative risks are in:


$$\begin{array}{r} RRO_{3}\left(27\;ug/m3\right)=\exp\left(0.007\right)=1.0\;visits\;per\;O_{3}\;unit\\ RRO_{3}\left(87ug/m3\right)=\exp\left(-0.045\right)=0.95\;visits\;per\;O_{3}unit \end{array}$$

**Table 3:** As a result of the best model for asthma, the sign in the visits column means an increment (+) or reduction (–) in the number of visits.

**Variable**	**Estimate**	**(Pr > t) < 0.05**	**Visits**
Age 0–5	1.94	^*^	+
Age 19–35	2.29	^*^	+
Age 36–50	2.37	^*^	+
Age 51–65	2.16	^*^	+
Age 66–100	1.66	^*^	+
Monday	0.52	^*^	+
Tuesday	0.43	^*^	+
Wednesday	0.46	^*^	+
Thursday	0.41	^*^	+
Friday	0.30	^*^	+
Saturday	0.03		-
August	0.11		-
July	0.20		-
June	0.07		-
May	0.06		+
November	0.05		+
September	0.27		+
March	0.06		-
December	0.10		-
February	0.26	^*^	-
January	0.25	^*^	-
October	0.24		+
Lag day 1	1.52	^*^	-
Lag day 2	0.26		-
Lag day 3	1.64	^*^	-
Lag day 4	1.05	^*^	+
Lag day 5	0.67	^*^	-
Lag day 6	1.23	^*^	-
Lag day 7	0.84	^*^	-
Lag day 8	1.09	^*^	-
Lag day 9	0.31	^*^	-
Lag day 10	0.76	^*^	+

NS, Statistically non-significant (*p* > 0.05), R^2^: 0.55. Smoother: s(O_3_). ^*^p-value < 0.05.

## Discussion

This study demonstrated a relationship between different environmental pollution components and increased medical consultations for respiratory diseases, asthma, and hypertension. Specifically O_3_ and NO_2_ were positively associated with the number of respiratory disease consultations, with the most affected age groups being 19–35 and over 66. For hypertension consultations, PM_2.5_ showed a more significant effect than O_3_ and NO_2_, and a stronger correlation was observed with increasing age. Asthma consultations followed a cyclical pattern with both PM_2.5_ and O_3_ across all age groups, though the correlation was weaker in the over 66 group.

The present study was conducted using data from the Mexican Social Security Institute population, because of the available and reliable data regarding medical care demand. During the study period, the eligible population for medical care through Social Security was almost half of Mexico City's total population (22). This suggests that our findings may be extrapolated to at least 50% of Mexico City's population and therefore to other cities with similar demographic and healthcare characteristics. By including consultations from all levels of medical care, namely in family medicine units, general hospitals, and high specialty hospitals, our results may be applicable to all levels of care.

The data recording is highly reliable because it is conducted under a social security institution. However, it requires individuals seeking medical attention to be registered and enrolled in the IMSS (Mexican Social Security Institute). This study collected information exclusively from the IMSS database. Within IMSS, qualified personnel register medical consultations in the database, strictly adhering to the criteria set by the International Classification of Diseases, further ensuring data quality.

The measurement of environmental pollution in Mexico City is standardized. As a result, the operation of monitoring equipment data collection, and information recording undergo constant review and validation. This standardization provides robust quality assurance, thereby ensuring reliable data on exposure to various environmental pollutants throughout Mexico City.

### Respiratory diseases

The strongest association was observed with O_3_ and NO_2_, indicating that these pollutants significantly impact. While PM_2.5_ showed a weaker association, it was still statistically significant.

Respiratory diseases, particularly bronchiolitis, are a leading cause of hospital admission in children under 2 years old. Although poor air quality affects everyone, children are disproportionately impacted due to their physiological and immune system immaturity. Their smaller airway caliber, higher metabolism, and greater respiratory rate compared to adults, lead to larger inhaled air volumes. This amplifies the effect of pollutants, overwhelming their limited capacity to neutralize and eliminate these environmental contaminants (23, 24). Consistent with this, studies have linked NO_2_, primarily from vehicle emissions in urban areas, to an increased incidence of lower respiratory tract infections and a higher risk of hospital admission for bronchiolitis (23, 24). Furthermore, Yamazaki et al., observed a positive association between PM_2.5_ and O_3_ concentrations and a reduced lung function in children (25).

For upper respiratory illnesses, our study found a positive correlation between these pathologies and elevated PM_2.5_, NO_2_, and O_3_ concentrations. These findings align with previous research; for instance, one study also reported a positive association between PM_2.5_ and the monthly case counts in outpatient consultations (26). Another article similarly linked daily PM_2.5_ concentrations to hospital admissions for upper respiratory tract infections in adults (27). It is believed that PM_2.5_ leads to inflammation and cell damage by producing free radicals and reactive oxygen species. Such inflammation and respiratory system damage could increase susceptibility to upper respiratory diseases (26).

Regarding O_3_ and NO_2_, a 2022 time-series study identified a positive association between short-term exposure to these pollutants and emergency department visits for respiratory diseases and upper respiratory infections (28). As O_3_ and NO_2_ are the two main oxidative gaseous pollutants in the atmosphere; the mechanisms and pathways through which these pollutants could affect the respiratory system include systemic oxidative injuries and airway inflammation, leading to bronchoconstriction and systemic inflammatory responses (28).

### Hypertension

In the present study, PM_2.5_ particles showed the strongest association with hypertension.

Our findings align with research demonstrating that PM_2.5_ is linked to an increment in clinic visits for hypertensive diseases (29).

Further support comes from investigations in Guangzhou, China, which identified a significant risk correlation between ambient PM_2.5_ exposure and outpatient clinic visits and hospitalizations for hypertension (25, 26). In that study, PM_2.5_ was the only pollutant to show a substantial effect. A similar pattern emerged from a research involving older adults, revealing a significant association with higher systolic blood pressure and both non-significant and significant results for diastolic blood pressure (30).

Several pathophysiological mechanisms can explain the association between high PM_2.5_ concentrations and the prevalence of hypertension. Key among these are systemic inflammatory and oxidative responses, which progressively impair vascular function and lead to arterial remodeling (4). Other proposed mechanisms include: an altered autonomic nervous system that mediate arterial vasoconstriction, systemic lipid peroxidation leading to the production of pro- inflammatory cytokines or an overstimulated sympathetic nervous system. This can enhance renin- angiotensin activity, improving myocardial contractility, and finally increasing blood pressure (29, 31).

### Asthma

Our study revealed stronger associations between asthma and both PM_2.5_ and O_3_ compared to previous research using the same Mexico City data (32). A notable distinction lies in our examination of various age groups where we found associations across all demographics. The strength of this association was weaker in individuals over 66 years old. This nuanced finding was not reported in our earlier article (32).

These findings are consistent with prior studies, which also linked increased ozone and PM_2.5_ levels to hospital visits for asthma exacerbations (33, 34).

Overall, our results align with the broader literature reporting an association between O_3_ and PM_2.5_ and acute asthma exacerbations. It is also widely recognized that air pollutant-induced asthma exacerbations are more prevalent in children than in adults (35).

From the physiopathological perspective, air pollutants primarily trigger an immune response mediated by oxidative stress. PM_2.5_ contains biological and organic compounds with oxidative stress effects, which cause damage to the airway's mucosal barrier and cellular epithelium. This triggers a type 2 inflammatory response leading to the release of proinflammatory cytokines and an increased flow of inflammatory cells. This activates various signaling pathways such as NF-κB or PI3K/Akt, driving inflammatory T2 cell activity and, consequently, airway hyperreactivity (36, 37).

Regarding O_3_, its mechanism generates an accumulation of reactive oxygen species (ROS) through lipid peroxidation of pulmonary surfactant factor and lipid membranes. This ROS accumulation activates the release of IL-1β, IL-6, IL-23, IL- 33, TNF-α, and TSLP, causing a proinflammatory cascade in the respiratory mucosa. Subsequent activation of the RORγt gene leads to the transcription of IL-17A and IL-22. IL-1, IL-17A, and IL-22 then induce neutrophil activation, prompting these active neutrophils to release more ROS. This cascade results in a high flow of inflammatory cells, representing a critical point in severe asthma exacerbations (38).

### Public health implications

In LATAM, 120 million people were reported to be exposed to pollution levels that exceed WHO guidelines, and consequently, 7 million deaths. Mexico, as a county of the global south, experiences high levels of contamination due to strong industrialization, caused by fossil-fuel generated energy, vehicular pollution, thermal power generation, industrial energy production, open burning of municipal or agricultural waste, and especially due to poor management by the government. In LATAM, Mexico is the country with the most deaths attributable to air pollution from burning fossil fuels (3). The global burden of disease reported an estimated 4.58 million deaths attributable to exposure to PM_2.5_ in 2017, in a study conducted in 195 countries and territories worldwide (1).

In Mexico, this problem has been addressed with the instauration of programs and policies. Exposure to ambient PM_2.5_ contributes to a high pollution linked death rate. The improvement of the Vehicle Verification Program, the control of ostensibly polluting vehicles in Mexico City, has reportedly improved the air quality since 2018, resulting in 9% decrease in PM_2.5_ particles, 4% in PM_10_ and nitrogen dioxide, 3% in sulfur dioxide, and 2% in carbon monoxide (39). The Government of Mexico started a program “PROAIRE” in 2017. It aims to reduce by 2030, 75% of SO_2_; 58% of PM_2.5_; 46% of VOCs; and 32% of NO_2_ from total emissions (39) through strategies like: air quality monitoring program, atmospheric environmental contingency programs, sentinel health units' program for evaluating health effects from air pollutants and an inventory of atmospheric pollutant emissions (40).

In other countries, policies have been implemented to control air pollution, thereby reducing the public health implications. In Global North countries like Europe and North America, the push for the manufacturing and use of electric vehicles and low-emission fuels has helped decrease emissions from burning fuel. Continuous improvement in public transport and the establishment of bike lanes and sidewalks have also improved air quality in urban areas (3). China stands as an example that improved air pollution regulation policies lead to significant improvements in public health. In 2013, the State Council implemented the Air Pollution Prevention and Control Action Plan (APPCAP), which was, at the time, the strictest air pollution regulation policy to date. This initiative resulted in 47,240 fewer deaths and 710,020 fewer years of life lost in 74 major cities in 2017 compared to 2013. Furthermore, APPCAP led to a 5.6% decrease in mortality attributable to PM_2.5_ in Beijing in 2018 compared to 2014 (41). These results highlight the importance of understanding the health implications of specific air pollution components and the necessity of implementing effective actions to diminish them. To protect public health, action-oriented research should inform and drive targeted interventions aimed at reducing population exposure to harmful air pollutants. Our findings offer critical insights into exposure-risk thresholds, which can be translated into evidence-based public health policies. Specifically, our exposure-response analyses demonstrate that relatively small increases in PM _2.5_ and O_3_ concentrations are associated with measurable adverse health impacts. These findings underscore the need for comprehensive air quality interventions, including emissions controls, the promotion of clean transportation, and the expansion of urban green spaces. In addition, efforts to improve access to healthcare services can help mitigate the health burden of air pollution and address disparities in exposure among vulnerable urban populations (42).

Currently, mobile applications make it easy to ascertain the levels of environmental pollutants in the air. This capability would allow decision-makers to guide the population to take greater precautions when pollutant levels rise to figures that can be associated with an exacerbation of cardiovascular and respiratory diseases.

The study has some limitations. It relied solely on data from individuals enrolled in the IMSS, which may exclude informal workers or those covered by other healthcare systems, thus limiting generalizability to the entire population.

These findings underscore the importance of designing educational strategies targeted at populations with asthma and hypertension to prevent acute episodes that could escalate into severe events, especially during periods of heightened pollutant levels. The most vulnerable populations to respiratory illnesses should also remain vigilant when pollutant levels exceed the thresholds established by Mexican regulations.

It is also critical to implement public health policies and educational interventions aimed at reducing exposure to harmful air pollutants. In several of the most polluted countries in the world, particularly in China, and now increasingly in Mexico, there is growing awareness of the negative effects of pollution, not only from an environmental standpoint but also as a major public health concern. This heightened awareness has led to greater action, with policies aimed at reducing atmospheric pollutant levels. However, as demonstrated in this study, these efforts remain insufficient, given the persistently high concentrations of environmental pollutants, often exceeding the levels recommended by the WHO, and their impact on the public healthcare system. There is still significant work to be done in Mexico to meet the targets set for 2030.

Strengthening environmental regulations to maintain pollutant levels below national and international thresholds could significantly reduce the burden of preventable cardiorespiratory diseases. Future research should continue exploring these associations and assess the long-term effects of repeated exposure to atmospheric pollutants. It could also assess the impact of recent air quality regulations. Expanding the analysis to other Mexican cities or Latin American urban areas would also strengthen the regional understanding of pollution related health outcomes.

## Conclusion

There is a well-established global association between air pollution and both respiratory and cardiovascular diseases. This study provides strong evidence that short term exposure to elevated concentrations of atmospheric pollutants, specifically PM_2.5_, O_3_, and NO_2_, are significantly associated with an increase in medical visits for upper respiratory diseases, asthma, and hypertension in Mexico City. By employing Generalized Additive Mixed Models and analyzing health records from IMSS-affiliated hospitals between 2017 and 2019, we observed that the effects of air pollution varied by disease, age group, season, and day of the week. Respiratory diseases showed the strongest associations with O_3_ and NO_2_, particularly affecting individuals aged 19–35 and children under 5 years of age. Hypertension was predominantly linked to PM_2.5_, with older adults being the most affected. Asthma was related to PM_2.5_ and O_3_, affecting all age groups. A key strength of this study is its ability to provide insight into the public health burden of air pollution within an urban context with unique topographic and meteorological characteristics like Mexico City.

## Data Availability

The raw data supporting the conclusions of this article will be made available by the authors, without undue reservation.
