# Supplementary figures and images for: Association between daily variations in the levels of atmospheric O3, PM2.5, and NO2 and the frequency of hospital visits due to respiratory diseases and hypertension in Mexico City using Generalized Additive Mixed Models
Source: Front Public Health. 2025 Sep 2;13:1593285. doi: 10.3389/fpubh.2025.1593285 (PMC12436139; doi:10.3389/fpubh.2025.1593285)

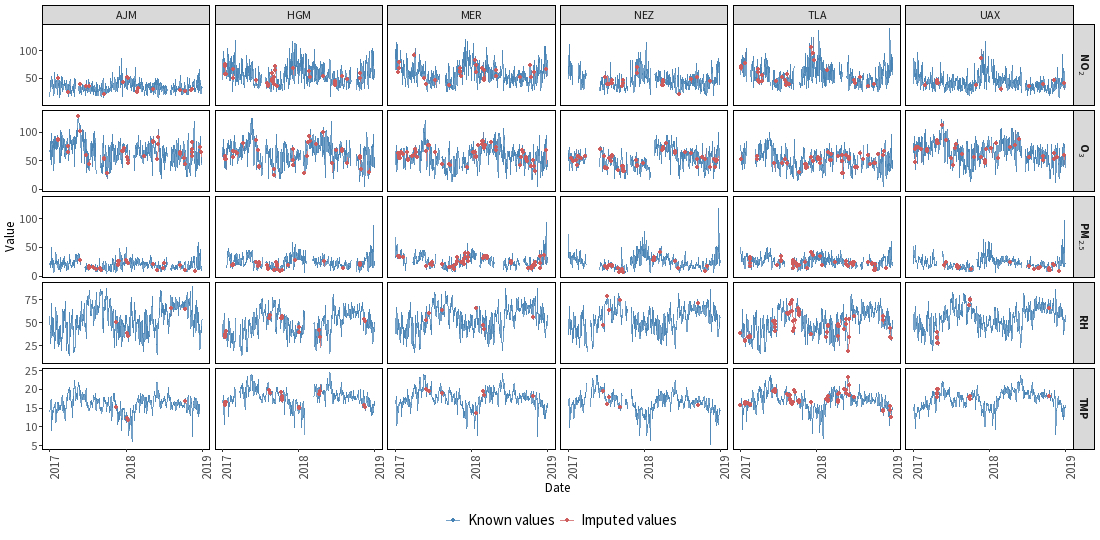

Supplement: Supplementary file 1 [file Image_1.jpg]
